# Supplementary material for: The Cancermuts software package for the prioritization of missense cancer variants: a case study of AMBRA1 in melanoma
Source: Cell Death Dis. 2022 Oct 15;13(10):872. doi: 10.1038/s41419-022-05318-2 (PMC9569343; doi:10.1038/s41419-022-05318-2)
Supplement: Supplementary file 4 — Supplemental table S4 [file 41419_2022_5318_MOESM4_ESM.docx]

**Supplemental table S4**

Multiple sequence alignment between the protein sequence of different AMBRA1 orthologs using Clustal Omega [Madeira, F., et al., *Search and sequence analysis tools services from EMBL-EBI in 2022.* Nucleic Acids Res, 2022.], using the protein sequences corresponding to the main Uniprot protein isoform of AMBRA1 from human (AMRA1_HUMAN), chimpanzee (A0A2I3TB21_PANTR), mouse (AMRA1_MOUSE), rat (F1LNQ2_RAT), bovine (F1MPW0_BOVIN), *Xenopus* (F6YH53_XENTR) and zebrafish ambra1a (AMR1A_DANRE).

CLUSTAL O(1.2.4) multiple sequence alignment

sp|E7FAG6|AMR1A_DANRE -MKLGQRNSVCILSSRERGAPGLASYRVLQQLVEEKTQRMKWQSQKVELPDSPRSTFLLA 59

tr|F6YH53|F6YH53_XENTR MKVVPEKNAVRILAGRERGLQAQGAQRLLQCLVEDKTRFMKWEGKKVELPDSPRSTFLLA 60

tr|F1MPW0|F1MPW0_BOVIN MKVVPEKNAVRILWGRERGTRTFGAQRLLQELVEDKTRCMKWEGKRVELPDSPRSTFLLA 60

sp|Q9C0C7|AMRA1_HUMAN MKVVPEKNAVRILWGRERGARAMGAQRLLQELVEDKTRWMKWEGKRVELPDSPRSTFLLA 60

tr|A0A2I3TB21|A0A2I3TB21_PANTR MKVVPEKNAVRILWGRERGARAMGAQRLLQELVEDKTRWMKWEGKRVELPDSPRSTFLLA 60

sp|A2AH22|AMRA1_MOUSE MKVVPEKNAVRILWGRERGTRAMGAQRLLQELVEDKTRWMKWEGKRVELPDSPRSTFLLA 60

tr|F1LNQ2|F1LNQ2_RAT MKVVPEKNAVRILWGRERGTRAMGAQRLLQELVEDKTRWMKWEGKRVELPDSPTLDFLLA 60

: ::*:* ** .**** .: *:** ***:**: ***:.::******* ****

sp|E7FAG6|AMR1A_DANRE FSPDRSLMASTHVNHNIYITEVKSGKCVHSLVGHRRTPWCLTFHPIIPGLIASGCLDGEV 119

tr|F6YH53|F6YH53_XENTR FSPDRTLLASTHVNHNIYITEVKTGRCIHSLVGHRRTPWCVTFHPTIPGLIASGCLDGEV 120

tr|F1MPW0|F1MPW0_BOVIN FSPDRTLLASTHVNHNIYITEVKTGKCVHSLIGHRRTPWCVTFHPTISGLIASGCLDGEV 120

sp|Q9C0C7|AMRA1_HUMAN FSPDRTLLASTHVNHNIYITEVKTGKCVHSLIGHRRTPWCVTFHPTISGLIASGCLDGEV 120

tr|A0A2I3TB21|A0A2I3TB21_PANTR FSPDRTLLASTHVNHNIYITEVKTGKCVHSLIGHRRTPWCVTFHPTISGLIASGCLDGEV 120

sp|A2AH22|AMRA1_MOUSE FSPDRTLLASTHVNHNIYITEVKTGKCVHSLIGHRRTPWCVTFHPTISGLIASGCLDGEV 120

tr|F1LNQ2|F1LNQ2_RAT FSPDRTLLASTHVNHNIYITEVKTGKCVHSLIGHRRTPWCVTFHPTISGLIASGCLDGEV 120

*****:*:***************:*:*:***:********:**** * ************

sp|E7FAG6|AMR1A_DANRE RIWDLHGGSESWLTESNSAIASLAFHPTAQLLLIATNNEVHLWDWSRKEPFTVVKTASET 179

tr|F6YH53|F6YH53_XENTR RIWDLHGGSESWFTDSNNAIASLAFHPTVQLLLIATASEIHFWDWSRREPFAVVKTASEM 180

tr|F1MPW0|F1MPW0_BOVIN RIWDLHGGSESWFTDSNNAIASLAFHPTAQLLLIATASEIHFWDWSRREPFAVVKTASEM 180

sp|Q9C0C7|AMRA1_HUMAN RIWDLHGGSESWFTDSNNAIASLAFHPTAQLLLIATANEIHFWDWSRREPFAVVKTASEM 180

tr|A0A2I3TB21|A0A2I3TB21_PANTR RIWDLHGGSESWFTDSNNAIASLAFHPTAQLLLIATANEIHFWDWSRREPFAVVKTASEM 180

sp|A2AH22|AMRA1_MOUSE RIWDLHGGSESWFTDSNNAIASLAFHPTAQLLLIATANEIHFWDWSRREPFAVVKTASEM 180

tr|F1LNQ2|F1LNQ2_RAT RIWDLHGGSESWFTDSNNAIASLAFHPTAQLLLIATANEIHFWDWSRREPFAVVKTASEM 180

************:*:**.**********.******* .*:*:*****:***:*******

sp|E7FAG6|AMR1A_DANRE ERVRLVRFDPLGHYLLTAIVNPSNQPNDDDPEIPMDSVEMPHLRQRSFLQSQPARRTPIL 239

tr|F6YH53|F6YH53_XENTR ERVRLVRFDPLGHYLLTAIVNPSNQQSDEESEIPLDSAEIPIYRQRSLLQTQPVRRTPLL 240

tr|F1MPW0|F1MPW0_BOVIN ERVRLVRFDPLGHYLLTAIVNPSNQQGDDEPEIPVDGAELSHYRQRALLQSQPVRRTPLL 240

sp|Q9C0C7|AMRA1_HUMAN ERVRLVRFDPLGHYLLTAIVNPSNQQGDDEPEIPIDGTELSHYRQRALLQSQPVRRTPLL 240

tr|A0A2I3TB21|A0A2I3TB21_PANTR ERVRLVRFDPLGHYLLTAIVNPSNQQGDDEPEIPIDGTELSHYRQRALLQSQPVRRTPLL 240

sp|A2AH22|AMRA1_MOUSE ERVRLVRFDPLGHYLLTAIVNPSNQQGDDEPEIPIDGTELSHYRQRALLQSQPVRRTPLL 240

tr|F1LNQ2|F1LNQ2_RAT ERVRLVRFDPLGHYLLTAIVNPSNQQGDDEPEIPIDGTELSHYRQRALLQSQPVRRTPLL 240

************************* .*:: ***:*..*: ***::**:**.****:*

sp|E7FAG6|AMR1A_DANRE HNFLHILTSRNSVPQAGGAHSASTDG----------------------------SSDSSG 271

tr|F6YH53|F6YH53_XENTR HNFLHMLSSRSSGTQAGEQPPVQDSATPSPPPPPPPPLPPPSNDSNQRVLYTRIRERS-P 299

tr|F1MPW0|F1MPW0_BOVIN HNFLHMLSSRSSCIQVGEQSTVQDPATPSPPPPPPQPST----ERPRTSAYIRLRQRVSY 296

sp|Q9C0C7|AMRA1_HUMAN HNFLHMLSSRSSGIQVGEQSTVQDSATPSPPPPPPQPST----ERPRTSAYIRLRQRVSY 296

tr|A0A2I3TB21|A0A2I3TB21_PANTR HNFLHMLSSRSSGIQVGEQSTVQDSATPSPPPPPPQPST----ERPRTSTYIRLRQRVSY 296

sp|A2AH22|AMRA1_MOUSE HNFLHMLSSRSSGIQVGEQSTVQDSATPSPPPPPPQPST----ERPRTSAYIRLRQRVSY 296

tr|F1LNQ2|F1LNQ2_RAT HNFLHMLSSRSSGIQVGEQSTVQDSATPSPPPPPPQPST----ERPRTSAYIRLRQRVSY 296

*****:*:**.* *.* .. . .

sp|E7FAG6|AMR1A_DANRE PYTLMCVQPLGMVCFCSRCSAARVPSPPDEDPS----DSASLEAQAHTFSSARTEPLQMS 327

tr|F6YH53|F6YH53_XENTR GVSTPCCQDPSMLCLCIRCSSARLSSPLFPHPEAPGAPSTSSGVTSTSFSPVQTEPSHSL 359

tr|F1MPW0|F1MPW0_BOVIN PT-AECCQHLGILCLCSRCSGTRVPSLL-PHQD--SVPPASARATTPSFSFVQTEPFHPP 352

sp|Q9C0C7|AMRA1_HUMAN PT-AECCQHLGILCLCSRCSGTRVPSLL-PHQD--SVPPASARATTPSFSFVQTEPFHPP 352

tr|A0A2I3TB21|A0A2I3TB21_PANTR PT-AECCQHLGILCLCSRCSGTRVPSLL-PHQD--SVPPASARATTPSFSFVQTEPFHPP 352

sp|A2AH22|AMRA1_MOUSE PTTVECCQHPGILCLCSRCAGTRVPSLL-PHQD--SVPPASARATTPSFSFVQTEPFHPP 353

tr|F1LNQ2|F1LNQ2_RAT PTTVECCQHPGILCLCSRCAGTRVPSLL-PHQD--SVPPASARATTPSFSFVQTEPFHPP 353

* * .::*:* **:.:*: * . . :* . : :** .:*** :

sp|E7FAG6|AMR1A_DANRE R---------FSVESRAANRSSAFSSVYG--GGSNMRNHSSSSGRRGVTGMAPVPHFRQH 376

tr|F6YH53|F6YH53_XENTR ERQQQQHQSSAQHDHGLLSRPSAFSSVHSSTAGNTLRNLSLGPTRRSLTG----QPSRY- 414

tr|F1MPW0|F1MPW0_BOVIN E-----QASSAPHDPGLLSRPSAFSTVQSSTAGNTLRNLSLGPPRRSLGGPLSGHPSRY- 406

sp|Q9C0C7|AMRA1_HUMAN E-----QASSTQQDQGLLNRPSAFSTVQSSTAGNTLRNLSLGPTRRSLGGPLSSHPSRY- 406

tr|A0A2I3TB21|A0A2I3TB21_PANTR E-----QASSTQQDQGLLNRPSAFSTVQSSTAGNTLRNLSLGPTRRSLGGPLSSHPSRY- 406

sp|A2AH22|AMRA1_MOUSE E-----QASSTQQDQGLLNRPSAFSTVQSSTAGNTLRNLSLGPTRRSLGGPLSSHPSRY- 407

tr|F1LNQ2|F1LNQ2_RAT E-----QASSTQQDQGLLNRPSAFSTVQSSTAGNTLRNLSLGPTRRSLGGPLSSHPSRY- 407

. : .* ****:* . .*..:** * . **.: * *

sp|E7FAG6|AMR1A_DANRE PPGREGGGRHPGADWTVSGL----NGQSSSMTPQRTGASSVSLLSVLRQQETSFQSPVYT 432

tr|F6YH53|F6YH53_XENTR --QQ-PSTDLAGSEWTRTVLSMGPRPEMEPMPPPRTSASSVSILSVLRQQEGETSSSVYT 471

tr|F1MPW0|F1MPW0_BOVIN --HRDIASGLTGSEWTRTVLSLNSRSEAESMPPPRTSASSVSLLSVLRQQEGGSQASVYT 464

sp|Q9C0C7|AMRA1_HUMAN --HREIAPGLTGSEWTRTVLSLNSRSEAESMPPPRTSASSVSLLSVLRQQEGGSQASVYT 464

tr|A0A2I3TB21|A0A2I3TB21_PANTR --HREIAPGLTGSEWTRTVLSLNSRSEAESMPPPRTSASSVSLLSVLRQQEGGSQASVYT 464

sp|A2AH22|AMRA1_MOUSE --HRELAPGLTGSEWTRTVLTLNSRSEVESMPPPRTSASSVSLLSVLRQQEGGSQASVYT 465

tr|F1LNQ2|F1LNQ2_RAT --HRELAPGLTGSEWTRTVLTLNSRSEVESMPPPRTSASSVSLLSVLRQQEGGSQASVYT 465

: . *::** : * . : . * * **.*****:******** .: ***

sp|E7FAG6|AMR1A_DANRE SASDRWGSTPGTSSSRHRPPEEEGQSSSSSIHSVLRCNLYRYFMDYEGTQDTVQPLDGSR 492

tr|F6YH53|F6YH53_XENTR SATEGRGFSSSES----DSSAAPPPVHPTTTRTELQCDLRRFFLEYDRLHELEPGAPGTT 527

tr|F1MPW0|F1MPW0_BOVIN SATEGRGFPASGLAAESDGGNGSSQNNSGSIRHELQCDLRRFFLEYDRLQELDQSLSGEA 524

sp|Q9C0C7|AMRA1_HUMAN SATEGRGFPASGLATESDGGNGSSQNNSGSIRHELQCDLRRFFLEYDRLQELDQSLSGEA 524

tr|A0A2I3TB21|A0A2I3TB21_PANTR SATEGRGFPASGLATESDGGNGSSQNNSGSIRHELQCDLRRFFLEYDRLQELDQSLSGEA 524

sp|A2AH22|AMRA1_MOUSE SATEGRGFPSSGLATESDGGNGSSQNNSGSIRHELQCDLRRFFLEYDRLQELDQSLSGET 525

tr|F1LNQ2|F1LNQ2_RAT SATEGRGFPSSGLATESDGGNGSSQNNSGNIRHELQCDLRRFFLEYDRLQELDQSLSGET 525

**:: * . . : *:*:* *:*::*: :: *

sp|E7FAG6|AMR1A_DANRE -QDQQTQEMLNNNMDPEQPGPSHYQS----PYSGENPPHSHMNRCRVCHNLFTYNQGSRR 547

tr|F6YH53|F6YH53_XENTR --QGQTHEMLNNNLEPEQPGPSHQPPQAHSGDSTSNQPRGHINRCRACHNLLTFNNDTLR 585

tr|F1MPW0|F1MPW0_BOVIN PQAQQAQEMLNNNLESERPGPSHQPT-PHSSENNSNLSRGHLNRCRACHNLLTFNNDTLR 583

sp|Q9C0C7|AMRA1_HUMAN PQTQQAQEMLNNNIESERPGPSHQPT-PHSSENNSNLSRGHLNRCRACHNLLTFNNDTLR 583

tr|A0A2I3TB21|A0A2I3TB21_PANTR PQTQQAQEMLNNNIESERPGPSHQPT-PHSSENNSNLSRGHLNRCRACHNLLTFNNDTLR 583

sp|A2AH22|AMRA1_MOUSE PQTQQAQEMLNNNIESERPGPSHLPT-PHSSENNSNLSRGHLNRCRACHNLLTFNNDTLR 584

tr|F1LNQ2|F1LNQ2_RAT PQTQQAQEMLNNNIESERPGPSHQPT-PHSSENNSNLSRGHLNRCRACHNLLTFNNDTLR 584

*::******:: *:***** . .* :.*:****.****:*:*:.: *

sp|E7FAG6|AMR1A_DANRE WDRTGQPS-STERNTPWQPSSSAFHSVA--PVSQSNEHLLEHRPIESTPNT--PEPHVPF 602

tr|F6YH53|F6YH53_XENTR WERTPPTYPETAGTSTWQPPPIPPPSSFQAGLAAGSTDHPGTPRVERVDLGSMAASSNRL 645

tr|F1MPW0|F1MPW0_BOVIN WERSTPNYSSGEASASWQVPGTF-----E-GMAASGSQLP---PLERTE-GQ-TASSSRL 632

sp|Q9C0C7|AMRA1_HUMAN WERTTPNYSSGEASSSWQVPSSF-----E-SVPSSGSQLP---PLERTE-GQ-TPSSSRL 632

tr|A0A2I3TB21|A0A2I3TB21_PANTR WERTTPNYSSGEASSSWQVPSSF-----E-SVPSSGSQLP---PLERTE-GQ-TPSSSRL 632

sp|A2AH22|AMRA1_MOUSE WERTTPNYSSGEASSSWHVSTTF-----E-GMPPSGNQLP---PLERTE-GQ-MPSSSRL 633

tr|F1LNQ2|F1LNQ2_RAT WERTTPNYSSGEASSSWHVSTTF-----E-GMPPSGNQLP---PLERTE-GQ-MPNSSRL 633

*:*: . .: *: : .. . :* . :

sp|E7FAG6|AMR1A_DANRE SQRTDTGQHEEQAVGLVFNQETGQLERVYRQSAS-SRSANISQGALNQEMPEDTPDNDYL 661

tr|F6YH53|F6YH53_XENTR ---ENGTTQEERTVGVVYNPETGHWERVYSQPASVSRAPNVSQEALPQDLQEESTEEDSL 702

tr|F1MPW0|F1MPW0_BOVIN ELGNSASPQEERTVGVAFNQETGHWERIYTQA---SRSGTVSQEALHQDLPEESSEEDSL 689

sp|Q9C0C7|AMRA1_HUMAN ELSSSASPQEERTVGVAFNQETGHWERIYTQS---SRSGTVSQEALHQDMPEESSEEDSL 689

tr|A0A2I3TB21|A0A2I3TB21_PANTR ELSSSASPQEERTVGVAFNQETGHWERIYTQS---SRSGTVSQEALHQDMPEESSEEDSL 689

sp|A2AH22|AMRA1_MOUSE ELSSSASSQEERTVGVAFNQETGHWERIYTQS---SRSGTVSQEALHQDMPEESSEEDSL 690

tr|F1LNQ2|F1LNQ2_RAT ELSSSASSQEERTVGVAFNQETGHWERIYTQS---SRSGTVSQEALHQDMPEESSEEDSL 690

. :**::**:.:* ***: **:* * **: .:** ** *:: *:: ::* *

sp|E7FAG6|AMR1A_DANRE R-----------------------------RLSPAAYYAQRMIQYLSRRDSVRQHSHRPP 692

tr|F6YH53|F6YH53_XENTR RRRLLESSLISLSRYDATGSREHPIYPDPARLSPAAYYAQRMIQYLSRRDSIRQRSMRYQ 762

tr|F1MPW0|F1MPW0_BOVIN R-----------------------------RLSPAAYYAQRMIQYLSRRDSIRQRSMRYQ 720

sp|Q9C0C7|AMRA1_HUMAN RRRLLESSLISLSRYDGAGSREHPIYPDPARLSPAAYYAQRMIQYLSRRDSIRQRSMRYQ 749

tr|A0A2I3TB21|A0A2I3TB21_PANTR RRRLLESSLISLSRYDGAGSREHPIYPDPARLSPAAYYAQRMIQYLSRRDSIRQRSMRYQ 749

sp|A2AH22|AMRA1_MOUSE RRRLLESSLISLSRYDGAGSREHPIYPDPARLSPAAYYAQRMIQYLSRRDSIRQRSMRYQ 750

tr|F1LNQ2|F1LNQ2_RAT RRRLLESSLISLSRYDGAGSREHPIYPDPARLSPAAYYAQRMIQYLSRRDSIRQRSMRYQ 750

* *********************:**:* *

sp|E7FAG6|AMR1A_DANRE SRPRPLSSNPSSLSPSPVPNAESSEVDFEEFEENGSR--YRTPRNARMSAPSLGRFVGTR 750

tr|F6YH53|F6YH53_XENTR QNR-LRTSAVSSSSEAQGA-VETGDLEYEEFEDNGDRSRHRTTRNARMSAPSLGRFVP-R 819

tr|F1MPW0|F1MPW0_BOVIN QNR-LRSSTSSSSSDNQGPTVEGTDLEFEDFEDSGDRSRHRAPRNARMSAPSLGRFVP-R 778

sp|Q9C0C7|AMRA1_HUMAN QNR-LRSSTSSSSSDNQGPSVEGTDLEFEDFEDNGDRSRHRAPRNARMSAPSLGRFVP-R 807

tr|A0A2I3TB21|A0A2I3TB21_PANTR QNR-LRSSTSSSSSDNQGPSVEGTDLEFEDFEDNGDRSRHRAPRNARMSAPSLGRFVP-R 807

sp|A2AH22|AMRA1_MOUSE QNR-LRSSTSSSSSDNQGPSVEGTDLEFEDFEDNGDRSRHRAPRNARMSAPSLGRFVP-R 808

tr|F1LNQ2|F1LNQ2_RAT QNR-LRSSTSSSSSDNQGPSVEGTDLEFEDFEDNGDRSRHRAPRNARMSAPSLGRFVP-R 808

.. :* ** * .* ::::*:**:.*.* :*: ************** *

sp|E7FAG6|AMR1A_DANRE RFLLPEFLPYAGIFHERGQPGLATHSSVNRVLAGAVIGDGQSAVASNIANTTYRLQWWDF 810

tr|F6YH53|F6YH53_XENTR RFLLPEYLPYAGIFHERGQPGLATHSSVNRVLAGAVIGDGQSAVASNIANTTYRLQWWDF 879

tr|F1MPW0|F1MPW0_BOVIN RFLLPEYLPYAGIFHERGQPGLATHSSVNRVLAGAVIGDGQSAVASNIANTTYRLQWWDF 838

sp|Q9C0C7|AMRA1_HUMAN RFLLPEYLPYAGIFHERGQPGLATHSSVNRVLAGAVIGDGQSAVASNIANTTYRLQWWDF 867

tr|A0A2I3TB21|A0A2I3TB21_PANTR RFLLPEYLPYAGIFHERGQPGLATHSSVNRVLAGAVIGDGQSAVASNIANTTYRLQWWDF 867

sp|A2AH22|AMRA1_MOUSE RFLLPEYLPYAGIFHERGQPGLATHSSVNRVLAGAVIGDGQSAVASNIANTTYRLQWWDF 868

tr|F1LNQ2|F1LNQ2_RAT RFLLPEYLPYAGIFHERGQPGLATHSSVNRVLAGAVIGDGQSAVASNIANTTYRLQWWDF 868

******:*****************************************************

sp|E7FAG6|AMR1A_DANRE TKFDLPEISNASVNVLVPNCKIYNDASCDISADGQLLAVFIPSSQRGFPDEGILAVYSLA 870

tr|F6YH53|F6YH53_XENTR TKYDLPEISNASINVLVQNCKIYNDASCDISADGQLLAAFIPSSQRGFPDEGILAVYSLA 939

tr|F1MPW0|F1MPW0_BOVIN TKFDLPEISNASVNVLVQNCKIYNDASCDISADGQLLAAFIPSSQRGFPDEGILAVYSLA 898

sp|Q9C0C7|AMRA1_HUMAN TKFDLPEISNASVNVLVQNCKIYNDASCDISADGQLLAAFIPSSQRGFPDEGILAVYSLA 927

tr|A0A2I3TB21|A0A2I3TB21_PANTR TKFDLPEISNASVNVLVQNCKIYNDASCDISADGQLLAAFIPSSQRGFPDEGILAVYSLA 927

sp|A2AH22|AMRA1_MOUSE TKFDLPEISNASVNVLVQNCKIYNDASCDISADGQLLAAFIPSSQRGFPDEGILAVYSLA 928

tr|F1LNQ2|F1LNQ2_RAT TKFDLPEISNASVNVLVQNCKIYNDASCDISADGQLLAAFIPSSQRGFPDEGILAVYSLA 928

**:*********:**** ********************.*********************

sp|E7FAG6|AMR1A_DANRE PHNLGEMLYSKRFGPNAISVSLSPMGRYVMVGLASRRILLHQISDHMVAQVFRLQQPHAG 930

tr|F6YH53|F6YH53_XENTR PHNLGEILFTKRFGPNAISVSLSPMGRYVMVGLASRRILLHPSTEHMVAQVFRLQKPHGG 999

tr|F1MPW0|F1MPW0_BOVIN PHNLGEMLYTKRFGPNAISVSLSPMGRYVMVGLASRRILLHPSTEHMVAQVFRLQQAHGG 958

sp|Q9C0C7|AMRA1_HUMAN PHNLGEMLYTKRFGPNAISVSLSPMGRYVMVGLASRRILLHPSTEHMVAQVFRLQQAHGG 987

tr|A0A2I3TB21|A0A2I3TB21_PANTR PHNLGEMLYTKRFGPNAISVSLSPMGRYVMVGLASRRILLHPSTEHMVAQVFRLQQAHGG 987

sp|A2AH22|AMRA1_MOUSE PHNLGEMLYTKRFGPNAISVSLSPMGRYVMVGLASRRILLHPSTEHMVAQVFRLQQAHGG 988

tr|F1LNQ2|F1LNQ2_RAT PHNLGEMLYTKRFGPNAISVSLSPMGRYVMVGLASRRILLHPSTEHMVAQVFRLQQAHGG 988

******:*::******************************* ::**********: *.*

sp|E7FAG6|AMR1A_DANRE ETSMRRVFDVVYPMAPDQRRHVSINSARWLPDPGLGLAYGTNKGDLVICRPVDVHSDGSS 990

tr|F6YH53|F6YH53_XENTR ETSMRRVFNVLYPMPADQRRHVSINSARWLPEPGLGLAYGTNKGDLVICRPEAFDNVCDQ 1059

tr|F1MPW0|F1MPW0_BOVIN ETSMRRVFNVLYPMPADQRRHVSINSARWLPEPGLGLAYGTNKGDLVICRPEASNSGVEY 1018

sp|Q9C0C7|AMRA1_HUMAN ETSMRRVFNVLYPMPADQRRHVSINSARWLPEPGLGLAYGTNKGDLVICRPEALNSGVEY 1047

tr|A0A2I3TB21|A0A2I3TB21_PANTR ETSMRRVFNVLYPMPADQRRHVSINSARWLPEPGLGLAYGTNKGDLVICRPEALNSGVEY 1047

sp|A2AH22|AMRA1_MOUSE ETSMRRVFNVLYPMPADQRRHVSINSARWLPEPGLGLAYGTNKGDLVICRPEALNSGIEY 1048

tr|F1LNQ2|F1LNQ2_RAT ETSMRRVFNVLYPMPADQRRHVSINSARWLPEPGLGLAYGTNKGDLVICRPEALNSGIEY 1048

********:*:*** ***************:******************* .. .

sp|E7FAG6|AMR1A_DANRE TSE-HSERMFTINNGGGVGPSSSRSGDRAGSSRTDRRSRRDIGLMNGVGLQPQPPAASVT 1049

tr|F6YH53|F6YH53_XENTR FWEQMNEAILQ--------HNTPRSSERPGTSRASWRSDRDMGLINAIGLQPRNPTTSVT 1111

tr|F1MPW0|F1MPW0_BOVIN YWDQLNETVFTV-------HSSSRSSERPGTSRATWRTDRDMGLMNAIGLQPRNPTTSVT 1071

sp|Q9C0C7|AMRA1_HUMAN YWDQLNETVFTV-------HSNSRSSERPGTSRATWRTDRDMGLMNAIGLQPRNPATSVT 1100

tr|A0A2I3TB21|A0A2I3TB21_PANTR YWDQLNETVFTV-------HSNSRSSERPGTSRATWRTDRDMGLMNAIGLQPRNPATSVT 1100

sp|A2AH22|AMRA1_MOUSE YWDQLSETVFTV-------HSSSRSSERPGTSRATWRTDRDMGLMNAIGLQPRNPTTSVT 1101

tr|F1LNQ2|F1LNQ2_RAT YWDQLNETVFTV-------HSSSRSSERPGTSRATWRTDRDMGLMNAIGLQPRNPTTSVT 1101

: .* :: .. **.:* *:**: *: **:**:*.:****: *::***

sp|E7FAG6|AMR1A_DANRE SQGTQTQNQRLQHAETQTDRDLPDDPQQPSTSQG-SQVTDATESLDFETLPEDSGSEVVP 1108

tr|F6YH53|F6YH53_XENTR SQGTQTPAPQLQNAETQTEREVPESRPAPPTAEAGPSGTAETPSSSSEGA---------- 1161

tr|F1MPW0|F1MPW0_BOVIN SQGTQTLALQLQNAETQTEREIQEPGVAA----SGP------------------------ 1103

sp|Q9C0C7|AMRA1_HUMAN SQGTQTLALQLQNAETQTEREVPEPGTAA----SGP------------------------ 1132

tr|A0A2I3TB21|A0A2I3TB21_PANTR SQGTQTLALQLQNAETQTEREVPEPGTAA----SGP------------------------ 1132

sp|A2AH22|AMRA1_MOUSE SQGTQTLALQLQNAETQTEREEEEPGAAS----SGP------------------------ 1133

tr|F1LNQ2|F1LNQ2_RAT SQGTQTLALQLQNAETQTEREEEEPGTAS----SGP------------------------ 1133

****** :**:*****:*: : .

sp|E7FAG6|AMR1A_DANRE ETPPHSRPQEDEGSDPSEPSTDSTGQAEYVSRIRRLMAEGGMTAVVQREQSTTMASMGSF 1168

tr|F6YH53|F6YH53_XENTR ---PSSGESQEGIPSSSEVPGSGEGQEDALSRIQRLMAEGGMTAVVQREQSTTMASMGGF 1218

tr|F1MPW0|F1MPW0_BOVIN --------------GEGEGSDYGASGEDALSRIQRLMAEGGMTAVVQREQSTTMASMGGF 1149

sp|Q9C0C7|AMRA1_HUMAN --------------GEGEGSEYGASGEDALSRIQRLMAEGGMTAVVQREQSTTMASMGGF 1178

tr|A0A2I3TB21|A0A2I3TB21_PANTR --------------GEGEGSEYGASGEDALSRIQRLMAEGGMTAVVQREQSTTMASMGGF 1178

sp|A2AH22|AMRA1_MOUSE --------------GEGEGSEYGGSGEDALSRIQRLMAEGGMTAVVQREQSTTMASMGGF 1179

tr|F1LNQ2|F1LNQ2_RAT --------------GEGEGSEYGGSGEDALSRIQRLMAEGGMTAVVQREQSTTMASMGGF 1179

. .* . . : :***:************************.*

sp|E7FAG6|AMR1A_DANRE GNNIIVSHRIHRGSQTGADAQNRTRLSPIPGPSSGAPESLAAASYSRVLTNTLGFRGDTA 1228

tr|F6YH53|F6YH53_XENTR GNNIIVSHRIHRGSQTASDPASRASAPRSPQPSTSRETVAELE--------RLLAPPQTS 1270

tr|F1MPW0|F1MPW0_BOVIN GNNIIVSHRIHRSSQTGTEPGAA--HAPSPQPSTSRELLPEAG--------QLTERG--- 1196

sp|Q9C0C7|AMRA1_HUMAN GNNIIVSHRIHRSSQTGTEPGAA--HTSSPQPSTSRGLLPEAG--------QLAERG--- 1225

tr|A0A2I3TB21|A0A2I3TB21_PANTR GNNIIVSHRIHRSSQTGTEPGAA--HTSSPQPSTSRGLLPEAG--------QLAERG--- 1225

sp|A2AH22|AMRA1_MOUSE GNNIIVSHRIHRSSQTGTESGAA--RTSSPQPSTSRGLPSEPG--------QLAERA--- 1226

tr|F1LNQ2|F1LNQ2_RAT GNNIIVSHRIHRSSQTGTESGAA--RTSSPQPSTSRGLLSEPG--------QLAERG--- 1226

************.***.:: * **:. *

sp|E7FAG6|AMR1A_DANRE QGIDLTEQERLHTSFFTPEFSPLFSSAVDATGPSSSIGADSVLEGEDFHDFASLPPSLLS 1288

tr|F6YH53|F6YH53_XENTR Q-------------LLLPETQLLTNNNNDGEVN-----VQPLSSG--------------- 1297

tr|F1MPW0|F1MPW0_BOVIN ---------------LSPRTASWEQPATPGREP--------ALPSSS----PAPPPAHLP 1229

sp|Q9C0C7|AMRA1_HUMAN ---------------LSPRTASWDQPGTPGREP-----TQPTLPSSS----PVPIPVSLP 1261

tr|A0A2I3TB21|A0A2I3TB21_PANTR ---------------LSPRTASWDQPGTPGREP-----TQPTLPSSS----PVPIPVSLP 1261

sp|A2AH22|AMRA1_MOUSE ---------------LSPRTASWDQPSTSGREL-----PQPALSSSS----PVPIPVPLA 1262

tr|F1LNQ2|F1LNQ2_RAT ---------------LSPRTASWDQPSTSGREL-----PQPALSSSS----PVPIPVPLA 1262

: *. . . .

sp|E7FAG6|AMR1A_DANRE SS-----PSLSPVNNSNYSNSDSSYLGDEYGR------- 1315

tr|F6YH53|F6YH53_XENTR ----------------TFPGVE---R------------- 1304

tr|F1MPW0|F1MPW0_BOVIN STEGPTPPRCDLTNSNHLPDSGGSGRGEAAGPSGEPRDR 1268

sp|Q9C0C7|AMRA1_HUMAN SAEGPT-LHCELTNNNHLLDG-GSSRGDAAGPRGEPRNR 1298

tr|A0A2I3TB21|A0A2I3TB21_PANTR SAEGPT-LHCDLTNNNHLLDG-GSSRGDAAGPRGEPRNR 1298

sp|A2AH22|AMRA1_MOUSE SNEGPT-MHCNVTNNSHLPEGDGSNRGEAAGPSGEPQNR 1300

tr|F1LNQ2|F1LNQ2_RAT SNEGPT-MHCNVTNNSHLPEGDSSNVGEAAGPSGEPRNR 1300
