# Supplementary material for: The Cancermuts software package for the prioritization of missense cancer variants: a case study of AMBRA1 in melanoma
Source: Cell Death Dis. 2022 Oct 15;13(10):872. doi: 10.1038/s41419-022-05318-2 (PMC9569343; doi:10.1038/s41419-022-05318-2)
Supplement: Supplementary file 5 — Supplemental Figures [file 41419_2022_5318_MOESM5_ESM.pdf]

Supplemental Figure 1

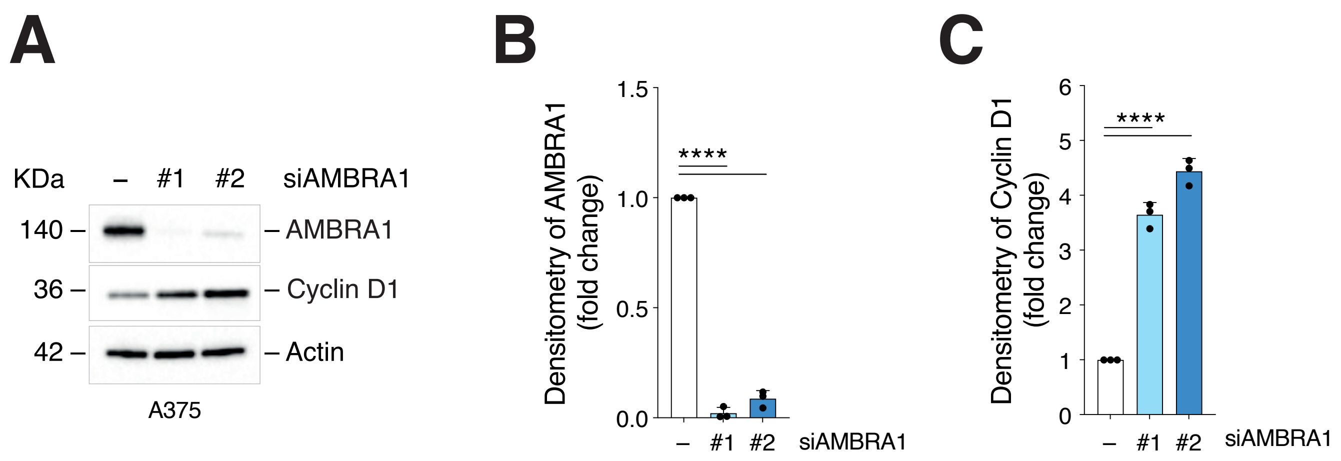

Supplemental Figure 1. Expression of Cyclin D1 upon AMBRA1 silencing in melanoma cells. (**A**) A375 cells were silenced for *AMBRA1* (siAMBRA1#1 and #2) and WB analyses performed to detect Cyclin D1 levels. AMBRA1 and Actin were used as transfection and loading control, respectively. Images are representative of n=3 independent experiments and are quantified in (**B**) for AMBRA1 and in (**C**) for Cyclin D1. Data are expressed as fold change ± SD vs control cells after normalization on Actin (n=3; ****p<0.0001; one-way ANOVA).

Supplemental Figure 2

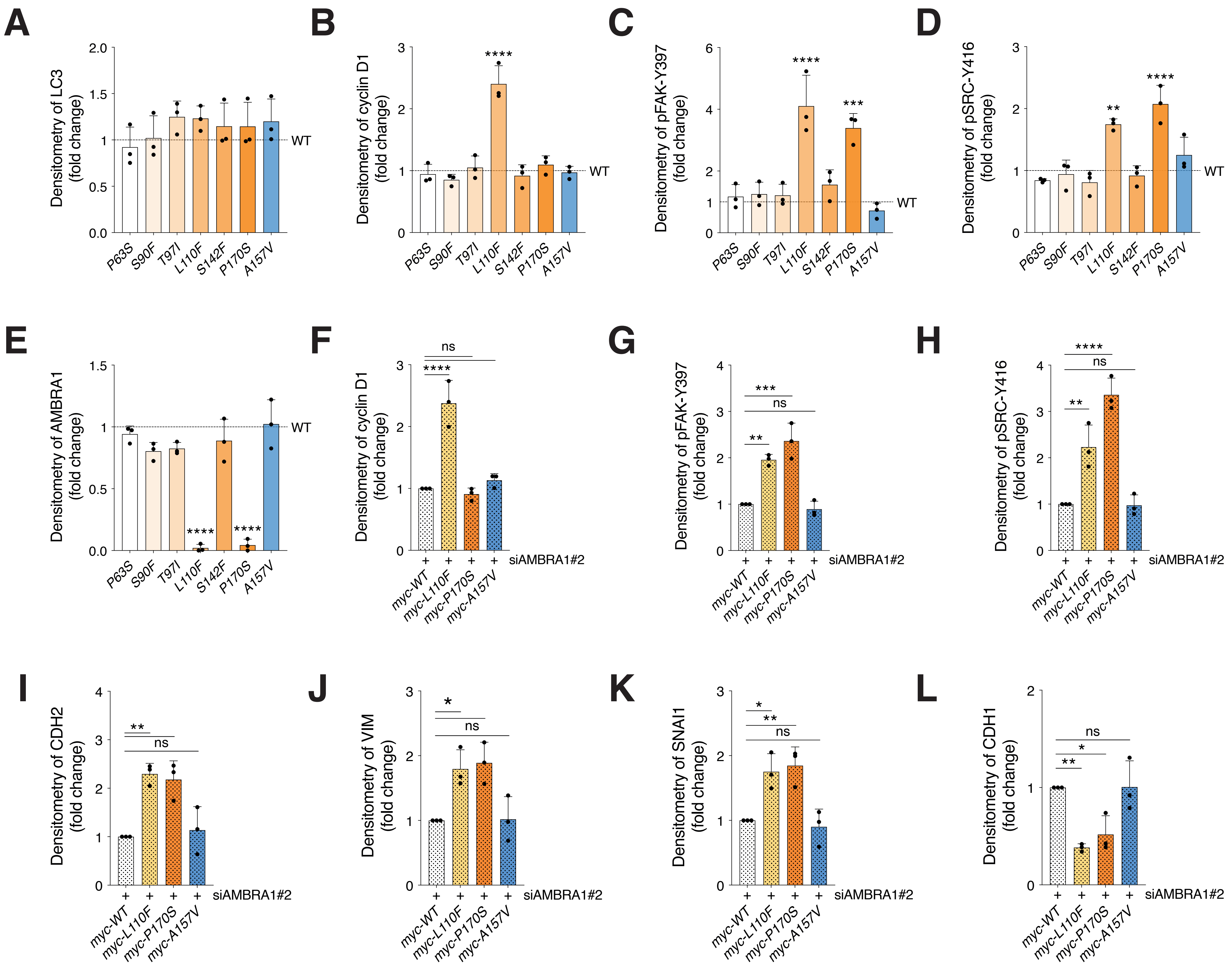

Supplemental Figure 2. Western Blot quantifications. (**A-E**) WB quantifications of A375 melanoma cells re-expressing the P63S, S90F, T97I, L110F, S142F and P170S AMBRA1 mutants. Quantifications are shown as fold change vs WT (represented by a dashed line) after normalization on the internal control Actin for (**A**) LC3 (LC3-II/LC3-I ratio), (**B**) for Cyclin D1 (****p<0.0001 vs WT; one-way ANOVA), (**C**) for pFAK-Y397 (pFAK-Y397/-FAK1 ratio) (***p=0.0001; ****p<0.0001 vs WT; one-way ANOVA), (**D**) pSRC-Y416 (pSCR-Y416/SRC ratio) (**p=0.0016; ****p<0.0001 vs WT; one-way ANOVA) and (**E**) for AMBRA1 (****p<0.0001 vs WT; one-way ANOVA). Data are expressed as mean ± SD (n=3). (**F-L**) WB quantifications of A375 melanoma cells re-expressing AMBRA1-myc-tagged WT, L110F, P170S and A157V constructs. Quantifications are shown as fold change vs myc-WT after normalization on the internal control Actin for (**F**) Cyclin D1 (****p<0.0001 vs myc-WT; one-way ANOVA), (**G**) pFAK-Y397 (pFAK-Y397/-FAK1 ratio) (**p=0.0021; ***p=0.0002 vs myc-WT; one-way ANOVA), (**H**) pSRC-Y416 (pSRC-Y416/SRC ratio) (**p=0.0047; ***p<0.0001 vs myc-WT; one-way ANOVA), (**I**) CDH2 (**p<0.0039 L110F vs myc-WT; **p<0.0069 P170S vs myc-WT; one-way ANOVA), (**J**) VIM (*p<0.0256 L110F vs myc-WT; *p<0.0142 P170S vs myc-WT; one-way ANOVA), (**K**) SNAI1 (*p=0.0166; **p<0.0085 vs myc-WT; one-way ANOVA), (L) CDH1 (*p=0.0228; **p<0.0058 vs myc-WT; one-way ANOVA). Data are expressed as mean ± SD (n=3).

Supplemental Figure 3

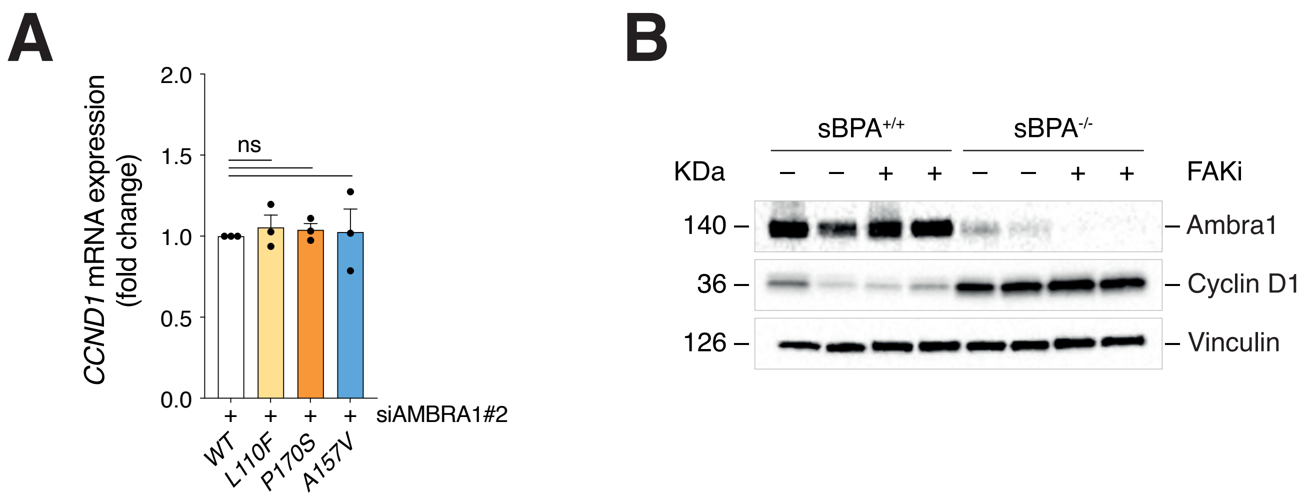

Supplemental Figure 3. Effect of mutants and FAKi on cyclin D1. (**A**) RT-qPCR analyses of *CCND1* upon WT, L110F, P170S and A157V re-expression. Data were normalized on *L34* and expressed as fold change vs AMBRA1 WT-transfected cells ± SEM (n=3; ns=not significant; one-way ANOVA). (**B**) WB analyses were performed on bulk tumors from sBPA+/+ (Ambra1 WT) and sBPA-/- (Ambra1 KO) mice treated with either Vehicle or 20mg/kg FAKi as described in Di Leo et al, 2021. The expression levels of Cyclin D1 were assessed; Ambra1 and Vinculin were detected as genotype and loading control, respectively. Two representative mice per group are shown.
