# Supplementary material for: The Cancermuts software package for the prioritization of missense cancer variants: a case study of AMBRA1 in melanoma
Source: Cell Death Dis. 2022 Oct 15;13(10):872. doi: 10.1038/s41419-022-05318-2 (PMC9569343; doi:10.1038/s41419-022-05318-2)

# Figure 3
## Panel C

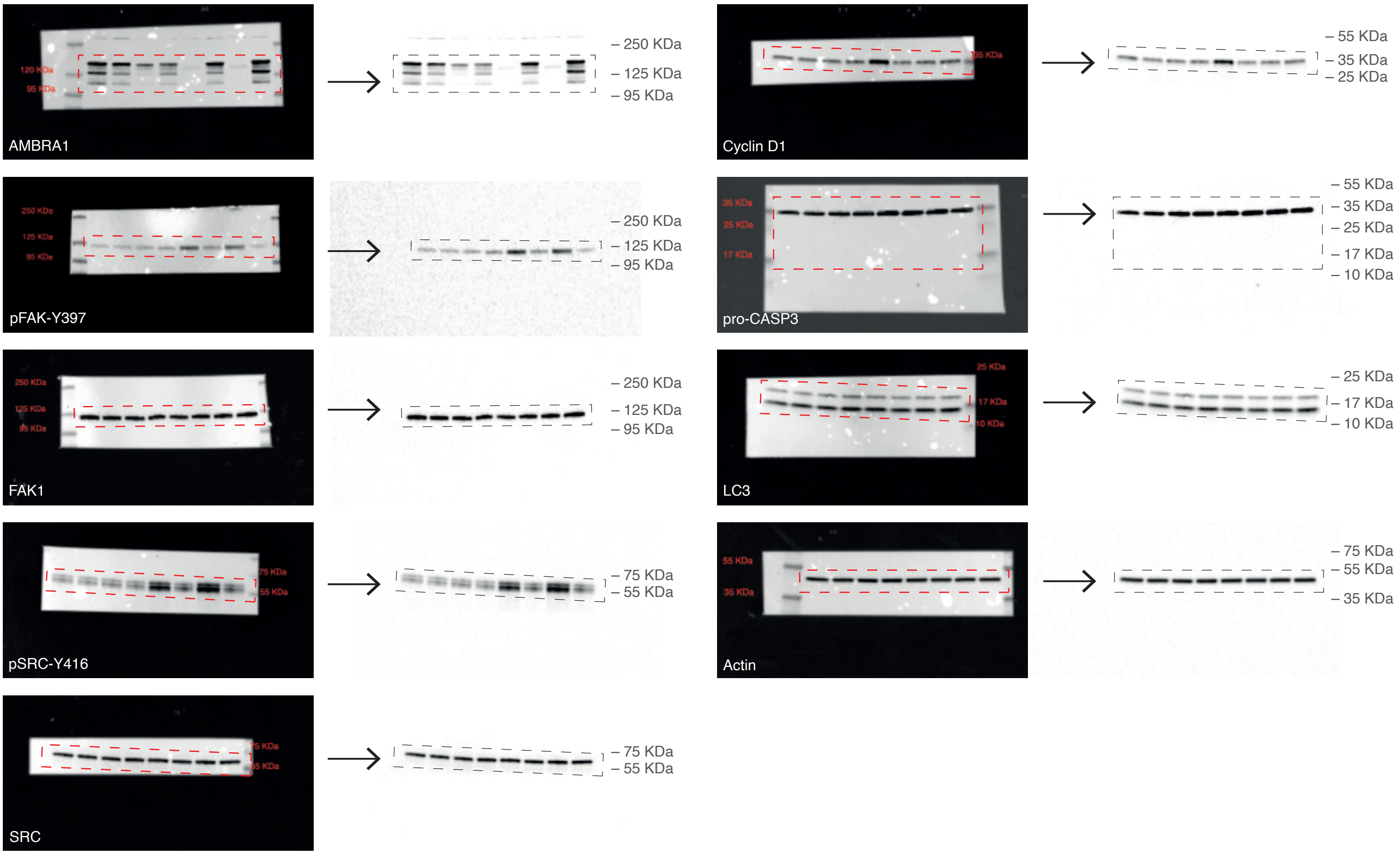


# Figure 4
## Panel B

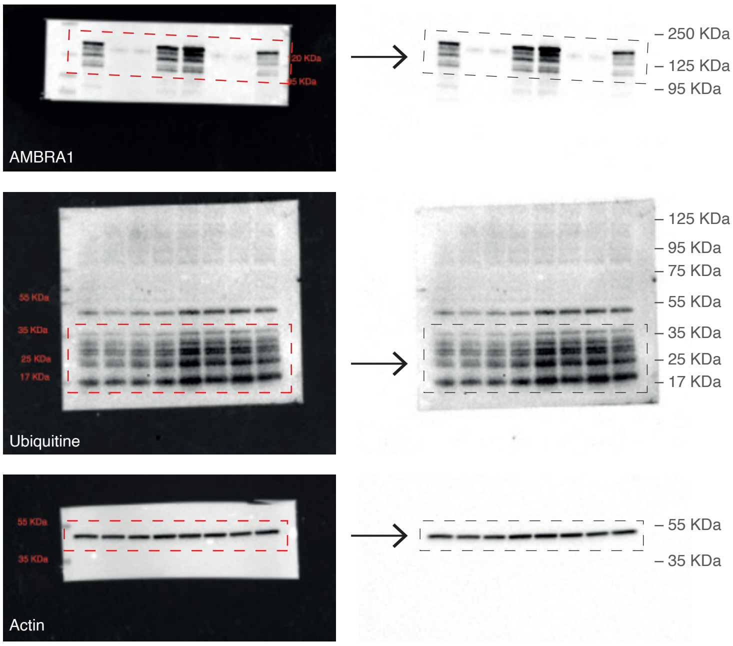


# Figure 4
## Panel C

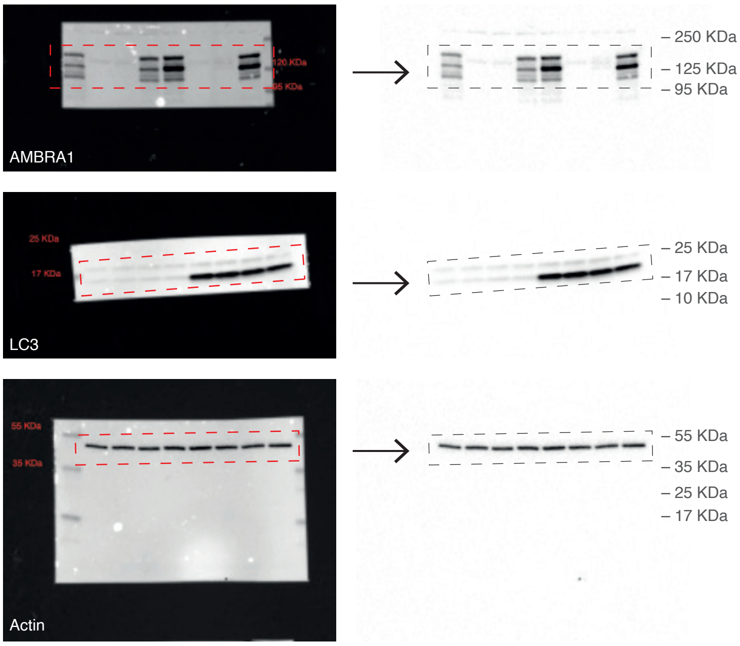

# Figure 4
## Panel D

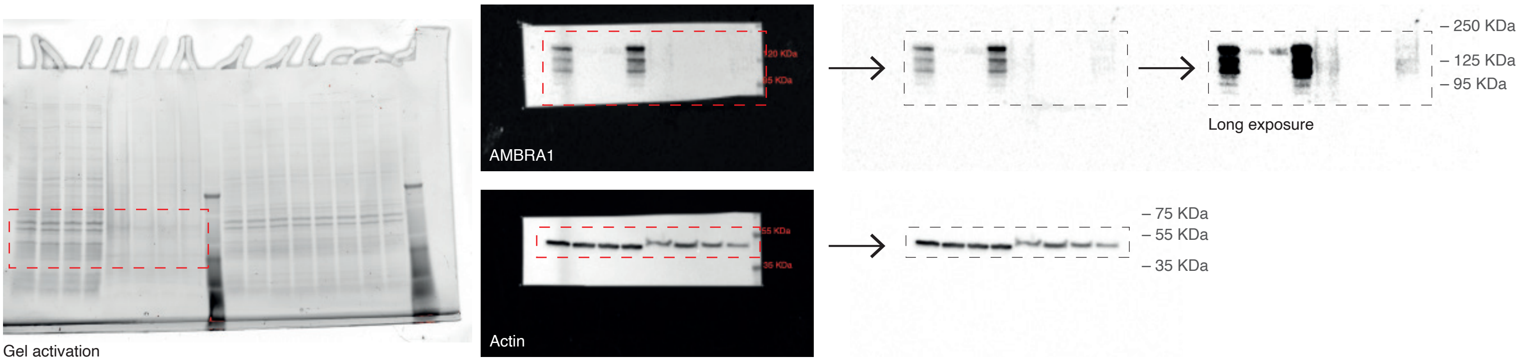


# Figure 4
## Panel E

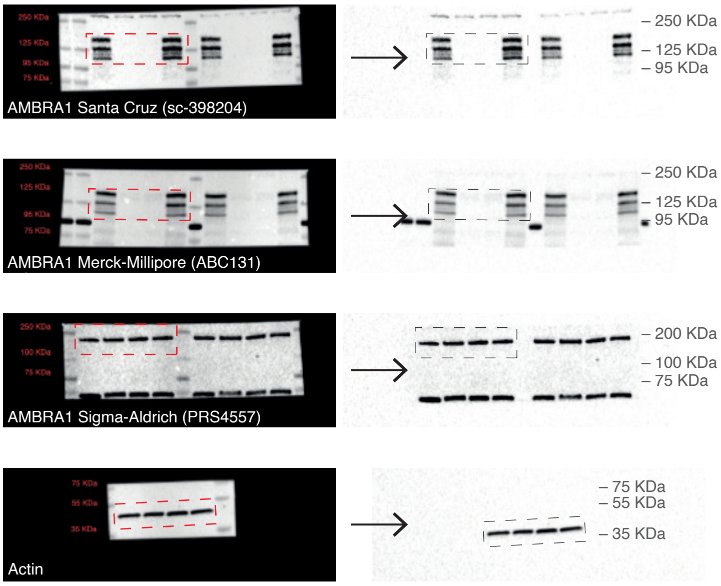


# Figure 4
## Panel F

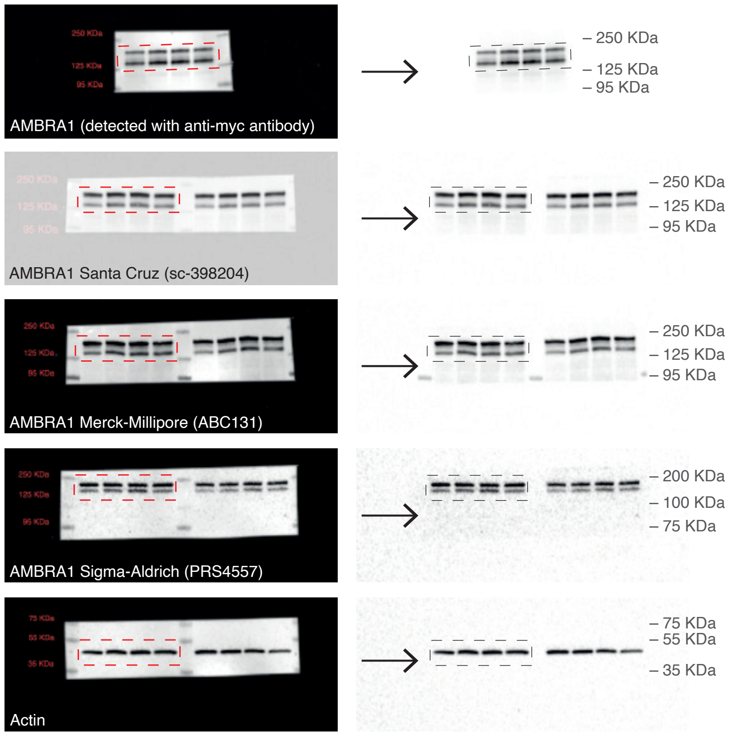


# Figure 4
## Panel G

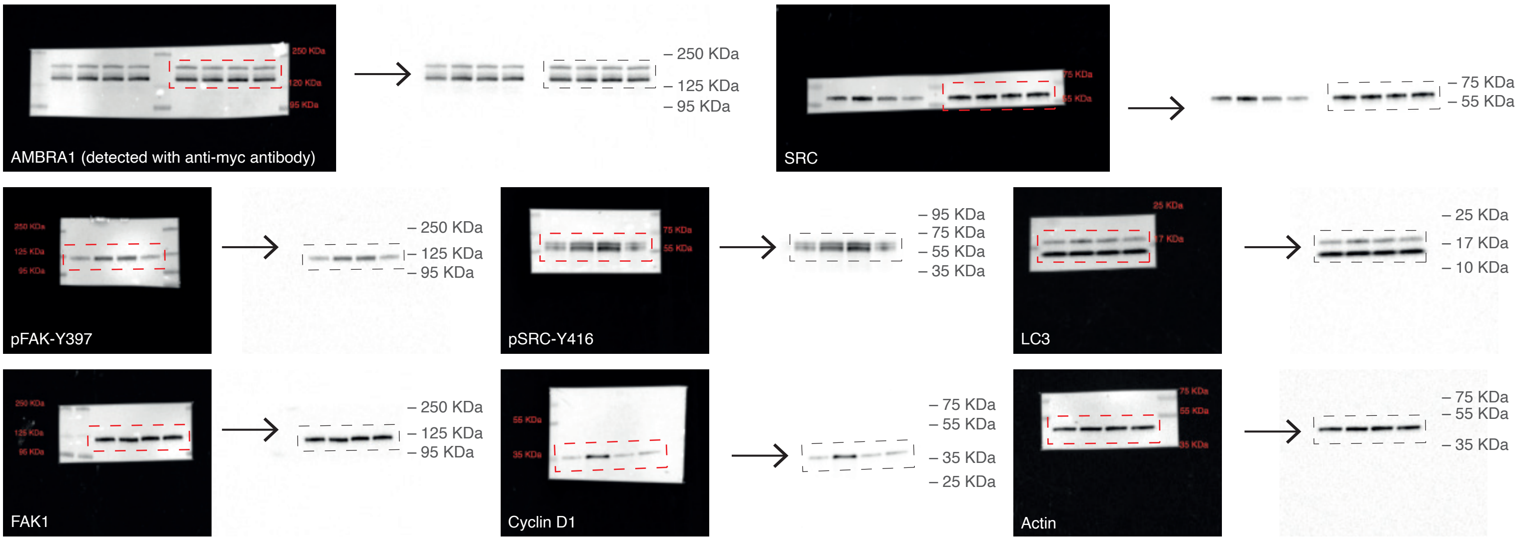

# Figure 5
## Panel H

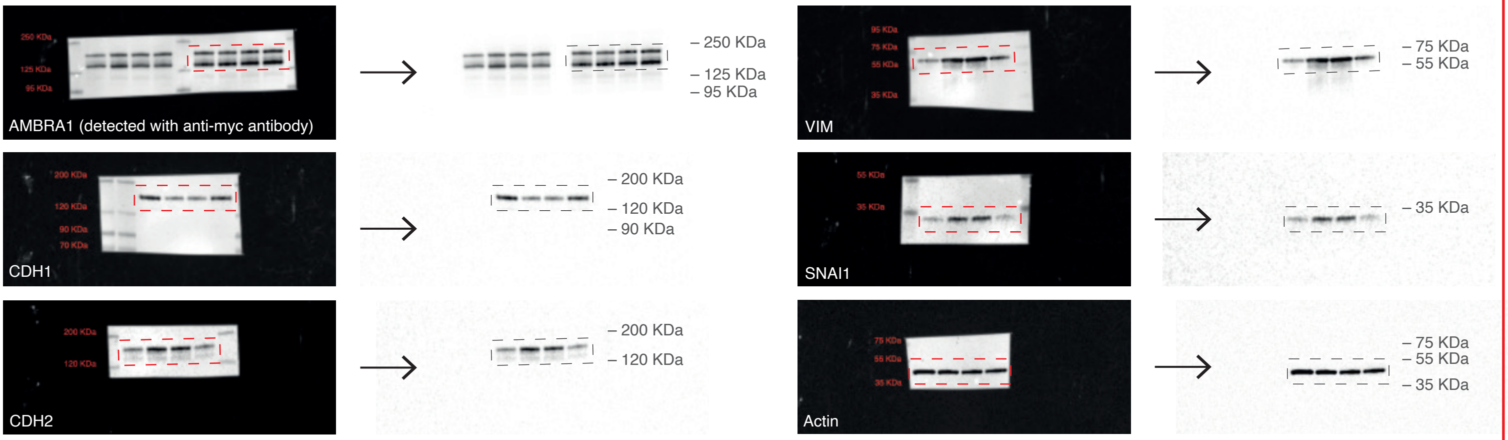


# Supplementary Figure 1
## Panel A

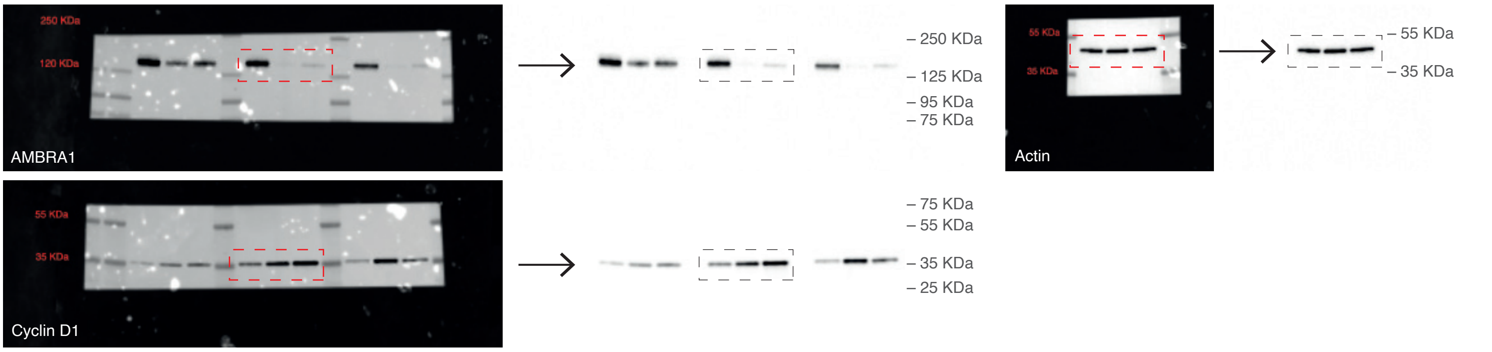


# Supplementary Figure 3
## Panel B

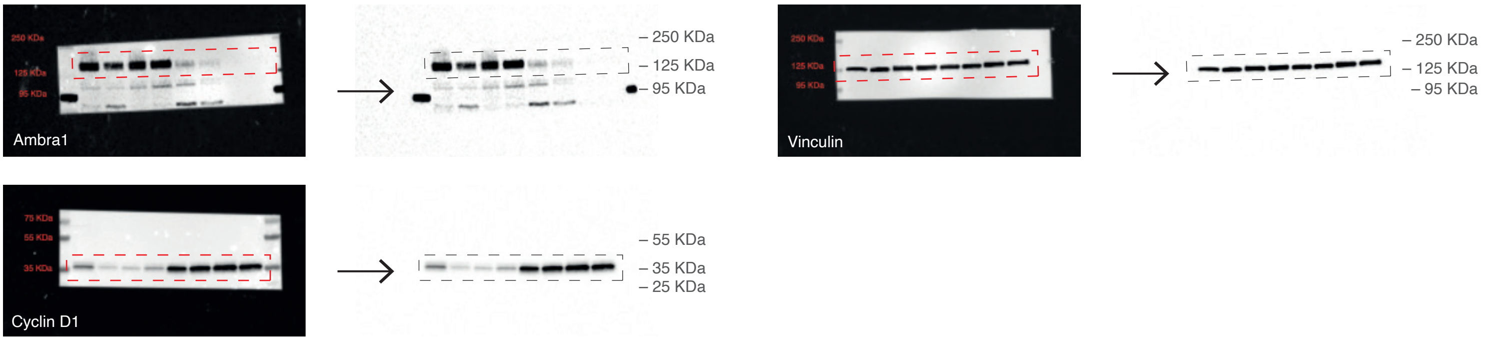

Supplement: Supplementary file 6 — Original Data File [file 41419_2022_5318_MOESM6_ESM.pdf]
